# Orthogonally modulated molecular transport junctions for resettable electronic logic gates

**DOI:** 10.1038/ncomms4023

**Published:** 2014-01-07

**Authors:** Fanben Meng, Yves-Marie Hervault, Qi Shao, Benhui Hu, Lucie Norel, Stéphane Rigaut, Xiaodong Chen

**Affiliations:** 1 School of Materials Science and Engineering, Nanyang Technological University, 50 Nanyang Avenue, Singapore 639798, Singapore; 2 Institut des Sciences Chimiques de Rennes, UMR 6226 CNRS—Université de Rennes 1, 263 Avenue du Général Leclerc, F-35042 Rennes cedex, France

## Abstract

Individual molecules have been demonstrated to exhibit promising applications as functional components in the fabrication of computing nanocircuits. Based on their advantage in chemical tailorability, many molecular devices with advanced electronic functions have been developed, which can be further modulated by the introduction of external stimuli. Here, orthogonally modulated molecular transport junctions are achieved via chemically fabricated nanogaps functionalized with dithienylethene units bearing organometallic ruthenium fragments. The addressable and stepwise control of molecular isomerization can be repeatedly and reversibly completed with a judicious use of the orthogonal optical and electrochemical stimuli to reach the controllable switching of conductivity between two distinct states. These photo-/electro-cooperative nanodevices can be applied as resettable electronic logic gates for Boolean computing, such as a two-input OR and a three-input AND-OR. The proof-of-concept of such logic gates demonstrates the possibility to develop multifunctional molecular devices by rational chemical design.

## Abstract (web summary)

Molecular transport junctions show promising applications in the fabrication of computing nanocircuits. Meng *et al*. design a family of organometallic compounds and use them in logic gates whereby molecular conductivity can be orthogonal and stepwise controlled by light and electrochemical potential.

## Graphical abstract

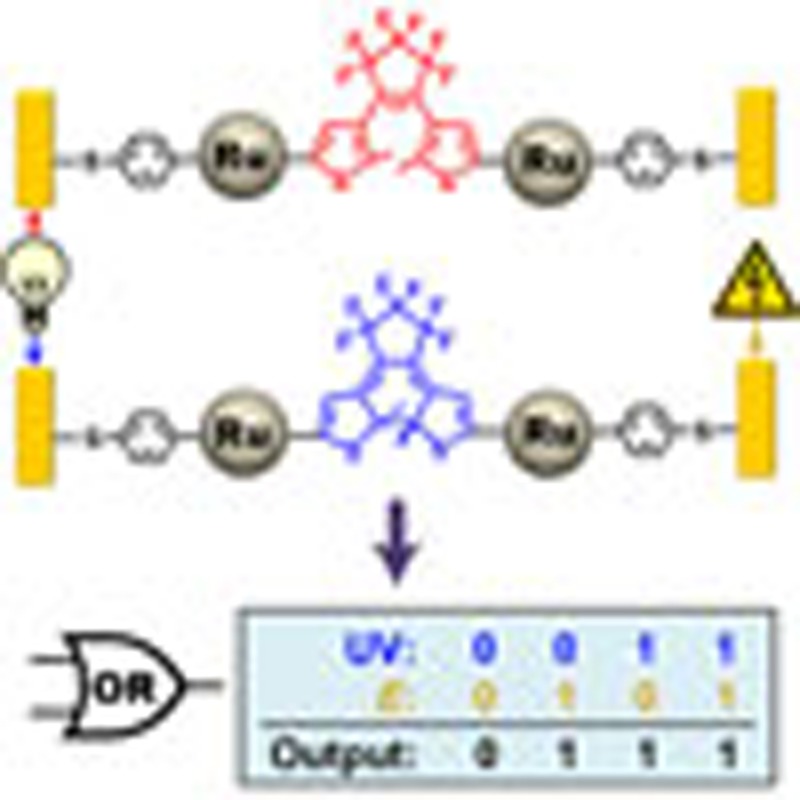

Since the first molecular rectifier proposed in 1974 (ref. 1), functional devices developed with individual molecules as functional components have exhibited promising applications in the fabrication of computing nanocircuits. Their advantages lie in synthetic tailorability, chemically controllable assembly, scalable size, and so forth^2^. As an essential structure in molecular electronics, molecular transport junctions (MTJs) have been used to investigate electronic conduction through functional molecules^3^,^4^,^5^,^6^. As a result, many electro-active properties, such as rectification^7^,^8^, negative differential resistance^9^,^10^ and Coulomb blockade^11^, have been uncovered, indicative of their promising applications in functional electronic devices. Additionally, external stimuli, such as light^12^,^13^,^14^,^15^,^16^, mechanical force^17^,^18^, magnetic field^19^,^20^, electrical field^21^,^22^,^23^ and electrochemical potential^24^,^25^,^26^,^27^,^28^, were introduced to modulate the molecular conductivity and thus to tune the properties of functional molecular devices, thereby significantly driving their development. As attested by the giant magnetoresistance effect^29^,^30^ and photoelectric cooperation^31^,^32^, a better synergy between such external stimuli and corresponding fabrication of multi-addressable MTJs may further efficiently advance the field of molecular electronics. Therefore, herein, we will take the advantages of synergetic modulation by multiple external controls, to explore multi-modulable molecular devices, which have not been addressed so far.

In this respect, chemical tailoring of functional molecules with multi-addressable properties will serve as an effective approach to fabricate such molecular devices. In order to address this issue, we designed and synthesized a new family of organometallic compounds, combining an ideal dithienylethene (DTE) photochromic unit^33^,^34^ and a unique electro-active carbon-rich ruthenium complex^35^,^36^,^37^,^38^,^39^, targeting multifunctional switching properties addressable with photon and/or electrons^40^,^41^,^42^,^43^. In a recent work^44^, we showed that the bimetallic compound **1** bearing two ruthenium fragments [AcS–C_6_H_4_–C≡C(dppe)_2_Ru]^+^ (dppe=1,2-bis(diphenylphosphino)ethane) on either ends of a DTE unit exhibits the reversible photo-controllable switching of conductivity when placed within a pair of gold nanoelectrodes fabricated by on-wire lithography (OWL)^45^,^46^,^47^. In such a molecular wire, the DTE unit undergoes photo-isomerization between a π-conjugated closed state (**c**) and a non-conjugated open state (**o**), and thus dominates the conductive state of the nanodevice, while the ruthenium moieties properly modulate electronic coupling between this DTE moiety and Au electrodes to avoid one-way switching^48^. In addition to this photochromic property, we previously showed that such a ruthenium/DTE association can also perform electrochemical cyclization of the DTE unit in solution at low potential^40^, in contrast to organic systems^49^,^50^. Therefore, since it has been successfully demonstrated that molecular conductance can be respectively tuned by light irradiation^12^,^13^,^14^,^15^,^16^,^44^ or electrochemical potential^24^,^25^,^26^,^27^,^28^, we were strongly motivated in the association of these two stimuli in a single electronic device to achieve orthogonally modulated MTJs.

In this contribution, we report for the first time the photo- and electro-commutation of MTJs based on OWL-generated nanogaps modified with two Ru/DTE complexes. First, the reversible and repeatable conductivity switching can be achieved upon orthogonal optical and/or electrochemical triggering of the molecular isomerization of **1o**, thanks to the remarkably low potential for electrochemical cyclization allowing the maintenance of stable thiol linking bonds at electrode interface. Then, a new longer trimetallic complex with two DTE units is designed towards a multi-addressable system and to further improve device complexity. It results in the fact that the corresponding device can achieve an original stepwise control of conductance requiring both stimuli. Finally, with a judicious choice of photo-/electro-inputs, OR and AND-OR Boolean computing are achieved within the orthogonally modulated MTJs.

## Results

### Optically and electrochemically co-modulated MTJs

**Figure 1:**
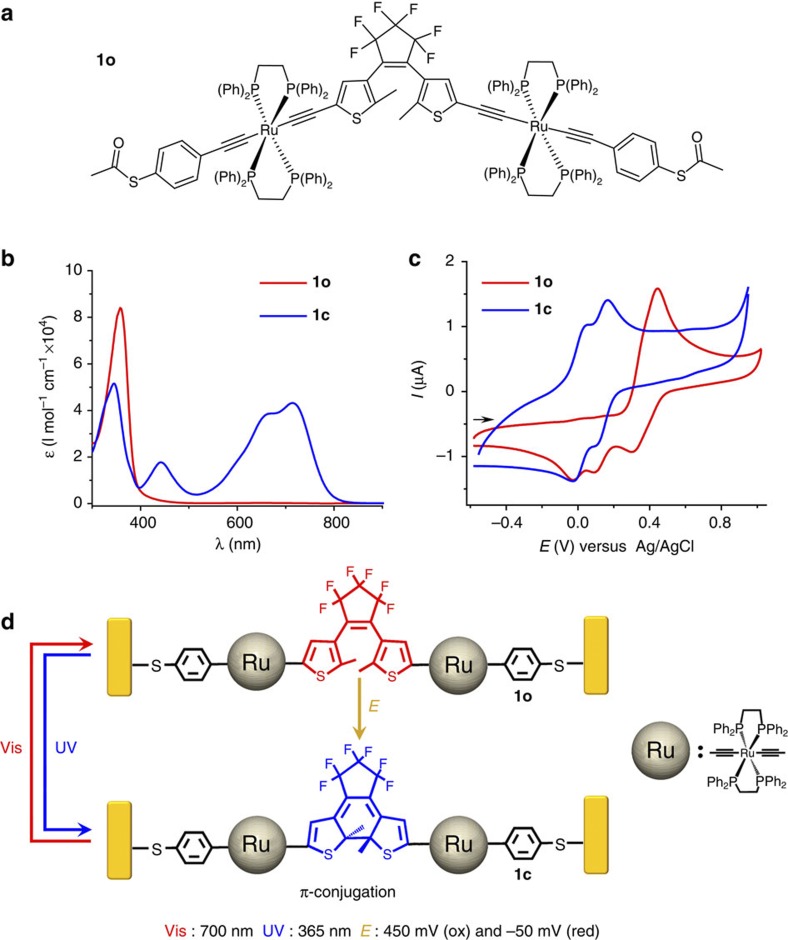
Orthogonally modulated isomerization of 1. (**a**) Chemical structure of **1o**. (**b**) Absorption spectra and spectral changes upon 350 nm irradiation of **1o** (50 μM in toluene). The process is fully reversible upon irradiation of **1c** at 750 nm. (**c**) CV of **1o** in CH_2_Cl_2_ (0.2 M Bu_4_NPF_6_) before (red line) and after irradiation at 350 nm leading to **1c** (blue line), scan rate 0.1 V s^−1^. (**d**) Scheme of molecular isomerization of **1** under external controls. E, electrolysis; UV, UV irradiation; Vis, visible light irradiation.

The binuclear compound **1** (Fig. 1a) can achieve photo-triggered isomerization both in solution and in solid-state nanodevices. As previously demonstrated^44^, reversible photochromic processes are achieved upon irradiation with UV light for ring closure and visible light for ring opening in solution (Fig. 1b). A typical cyclic voltammogram (CV) of **1o** is also shown on Fig. 1c. It presents a partially reversible wave at *E*_pa_=480 mV versus Ag/AgCl composed of two slightly separated one-electron oxidations for the two electronically independent metal fragments leading to **1o**^2+^. During the reverse cathodic scan, two new waves appear at less positive potentials and are ascribed to the **1c**^+^/**1c** and **1c**^2+^/**1c**^+^ processes involving the closed isomer electrochemically generated due to radical coupling as observed with its parent complex (Supplementary Fig. 1)^40^. Logically, identical waves are obtained after photolysis (*λ*=350 nm) of **1o**, that is, at *E°*_c1_=16 mV and *E°*_c2_=144 mV, along with the vanishing of the wave at ca. 480 mV. Therefore complex **1** is an ideal candidate to explore photo-/electro-switchable MTJs when incorporated into a pair of nanoelectrodes, as illustrated in Fig. 1d, which is also strongly supported by the theoretical calculations of molecular geometric reorganization and transmittance process through the molecule^44^.

**Figure 2:**
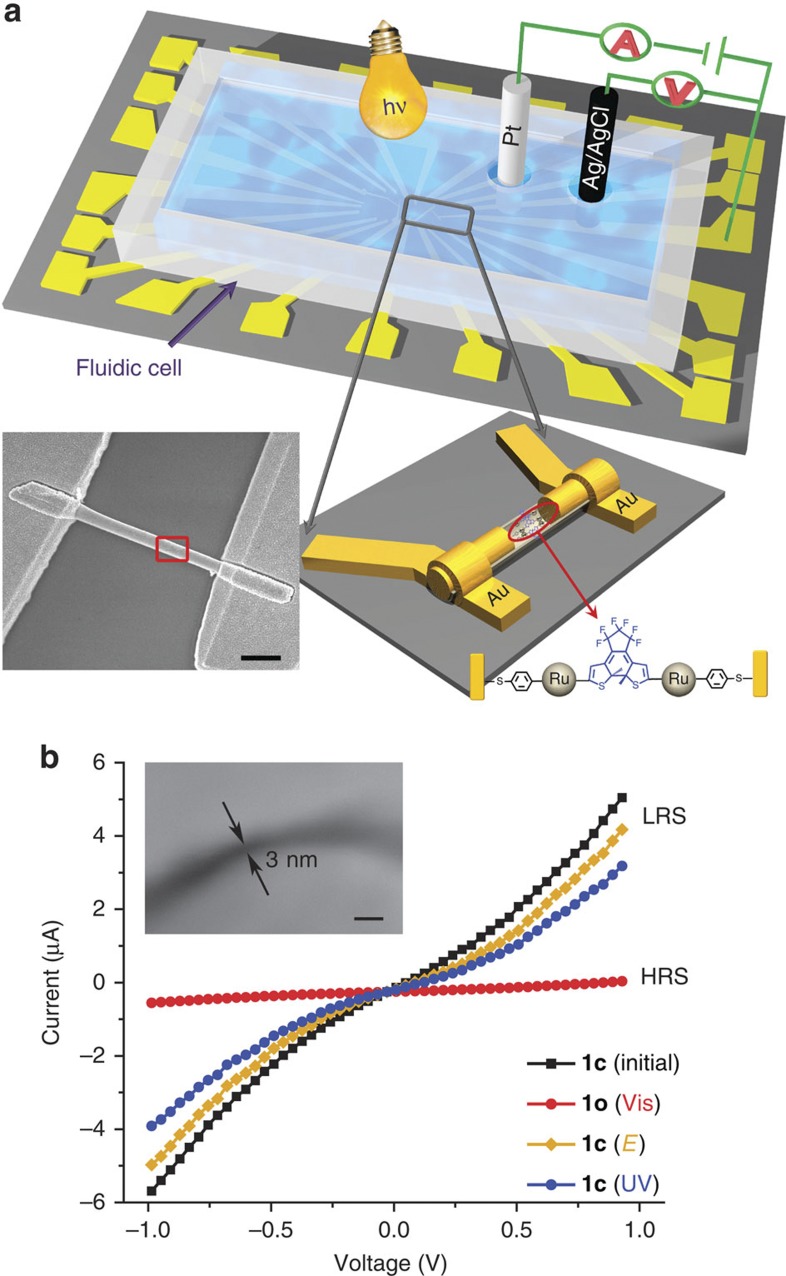
1c-functionalized nanogap devices. (**a**) Diagram of the functionalized nanogap devices. Inset: SEM image of a device fabricated by OWL-generated nanowire (scale bar: 1 μm). (**b**) *I–V* characteristics of ~3 nm gap devices loaded with **1c**, and obtained in the dark under vacuum. Black curve: initial **1c** device. Red curve: after 700 nm irradiation of **1c** device for 2 h to **1o** device. Yellow curve: after subsequent electrolysis of **1o** at 450 mV versus Ag/AgCl for 10 min followed by −50 mV for 10 min in CH_2_Cl_2_ to return to **1c** device. Blue curve: after 365 nm irradiation of **1o** device for 30 min to obtain the **1c** device. Inset: SEM image of a ~3 nm gap (scale bar: 10 nm).

OWL-generated gap-based nanodevices have been demonstrated as a unique testbed for molecular electronics^51^,^52^,^53^,^54^. In order to apply optical and electrochemical stimuli to functional molecules, the nanogap devices are further incorporated with one fluidic cell to achieve an electrochemical environment for device modulation, as shown in Fig. 2a. Electrochemical processes were carried out using a gold nanorod on one side of nanogap as the working electrode with a diameter of ~300 nm, a Pt wire as counter electrode and a homemade AgCl coated Ag wire as reference electrode (Fig. 2a). In a typical experiment, complex **1c** was covalently bonded onto the two sides of OWL-generated ~3 nm gaps (Fig. 2b, inset) after deprotection of the thiol, to ensure the accurate electronic coupling between the molecule and electrodes^51^,^54^. As expected, a photo-triggered conductive switching from low resistance state (LRS, Fig. 2b black curve) to high resistance state is observed (HRS, Fig. 2b, red curve) upon 700 nm irradiation, leading to the photochemical isomerization of π-conjugated **1c** to non-conjugated **1o**. The reverse process switches back the device to LRS via the photochemical closure upon UV light irradiation (365 nm) (Fig. 2b, blue curve).

Subsequently, electrochemical cyclization was also attempted with a device loaded with **1o** molecules by applying a positive potential of 450 mV versus Ag/AgCl followed by a slightly negative potential (−50 mV), in CH_2_Cl_2_ solution. For enabling comparison with photochemical processes, electrical measurements were then carried out in vacuum after removing CH_2_Cl_2_, in order to eliminate the influence of the solvent on the device conductivity and to only obtain information on molecular resistance (Supplementary Fig. 2). Thus, as attested by the resultant switching to the high conduction state (Fig. 2b, yellow curve), the electrochemical ring closure was successfully realized. By analogy with what occurs in solution, after applying a potential of 450 mV, it is anticipated that **1o** within the nanogap is oxidized to **1o**^**2+**^. Then, **1o**^**2+**^ undergoes radical coupling to form the more stable isomer **1c**^**2+**^, which is further reduced to **1c** by the slightly negative potential (−50 mV) applied subsequently. Importantly, due to the unique electronic structure of the ruthenium fragment, this electrochemical cyclization in the nanogap can be achieved at a low potential, avoiding the breakage of the Au–S bond, which is not possible in the case of pure organic DTE (>1 V potential is needed to perform ring closure)^49^,^50^. Moreover, this electrochemically obtained LRS state could be switched back to the initial HRS state (open state) upon visible light irradiation as described above.

### MTJ-based resettable electronic logic gates

**Figure 3:**
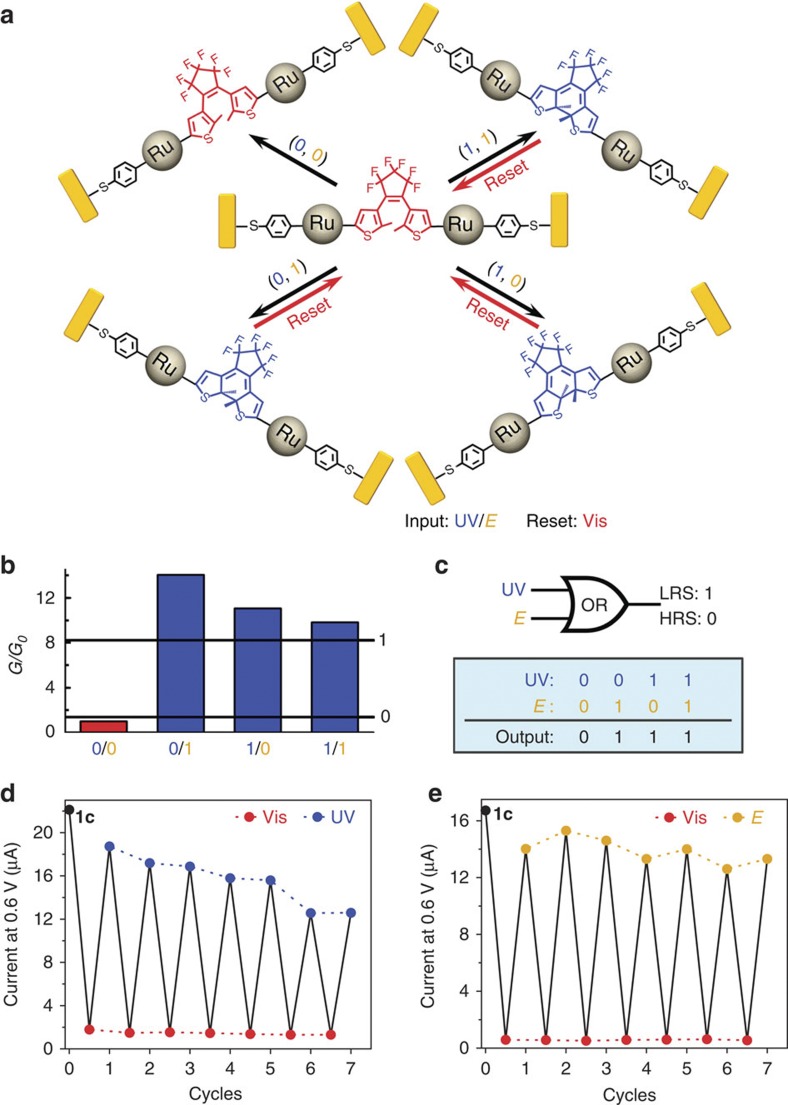
Resettable OR logic gate using 1c-based MTJs. (**a**) Scheme of **1**-based logic gate: **1** isomerization within nanogap devices under different inputs (UV irradiation and electrolysis). Reset: visible light irradiation (700 nm, 2 h). (**b**) Conductance (*G*) changes with different input combinations. (**c**) The electronic symbol and a truth table of the OR logic gate. (**d**,**e**) Endurance performance: current response of a **1c**-based nanodevice under alternate modulation of visible light irradiation (700 nm for 2 h) and (**d**) UV irradiation (365 nm for 30 min) or (**e**) Electrochemical stimuli (oxidation at 450 mV and reduction at −50 mV for 10 min). The current values were recorded at 0.6 V bias in vacuum.

Based on its unique orthogonally modulable property, the **1**-functionalized MTJs can be viewed as an electronic logic gate (Fig. 3a). The LRS **1c**-based device is first set to HRS (**1o**) by irradiation of 700 nm light. Then, the UV light irradiation and electrochemical stimulus are employed as inputs. The presence and absence of each input is respectively defined as ‘1’ and ‘0’. The outputs (‘1’ for LRS and ‘0’ for HRS) can be recorded by the conductance change from the previous HRS devices to the modulated devices. As shown in Fig. 3b, except in the case of absence of both inputs (0, 0), which leaves the device in HRS (output=0), the presence of either (0,1 or 1,0) or both inputs (1,1) could all trigger the molecular arrangement from the open state to the π-conjugated closed state, resulting in the enhancement of device conductivity to LRS (output=1). Hence, the Boolean computing of OR (Fig. 3c) has been achieved with the **1c**-functionlized nanodevices. In addition, a distinct advantage of such functional MTJs as logic gates is the resettability. Upon irradiation under visible light (700 nm), this nanodevice can be reset from LRS (1) to HRS (0). Thus, to illustrate the feasibility of these resettable MTJs as operative electronic devices, the reversibility and repeatability under the two different external stimuli have been investigated. As shown in Fig. 3d,e, the two distinct states of the device conductivity can be clearly observed after different cycles. The reversible photo- and electro-triggered resistive switching can be repeatedly operated for several cycles and it maintains a bi-state system, indicating the potential application of such a device in resettable electronic logic gates. As previously reported by us^44^ and others^12^,^13^,^14^,^15^,^16^, a progressively reduced reversibility in the course of a cycling test using the photochemical stimuli was observed (Fig. 3d). As the conductance of the devices showed no obvious change but rather just regular fluctuations with several electrochemical modulations (Fig. 3e, yellow dots), it can be concluded that UV irradiation is responsible for decomposition of some molecules and of the subsequent conductance drop (Fig. 3d, blue dots), rather than desorption of molecules from the gold surface due to stochastic behaviour of thiols^55^,^56^ or mechanical breaking of bonds upon isomerization as previously proposed. Consequently, the electrochemical control appears to be more reliable in maintaining a stable conductance. Another possible reason for the reduced reversibility, especially comparing with the initial device state, is that some molecules are connected in the nanogap with some constraints due to the roughness of the gap or interactions with other molecules. The first opening process can reduce these constraints and thus further re-closing is not favourable. Nevertheless, herein, an OR logic gate has been developed by the orthogonally modulated MTJs. In contrast to other logic gates based on chemical systems^57^,^58^,^59^,^60^, such molecular nanodevices exhibit unique advantages of resettablity^61^,^62^. Additionally, direct electronic outputs will be more convenient for nanocircuit construction compared with other outputs such as optical signals that have arisen from a similar complex^43^,^63^.

### Multi-addressable MJTs for advanced Boolean computing

**Figure 4:**
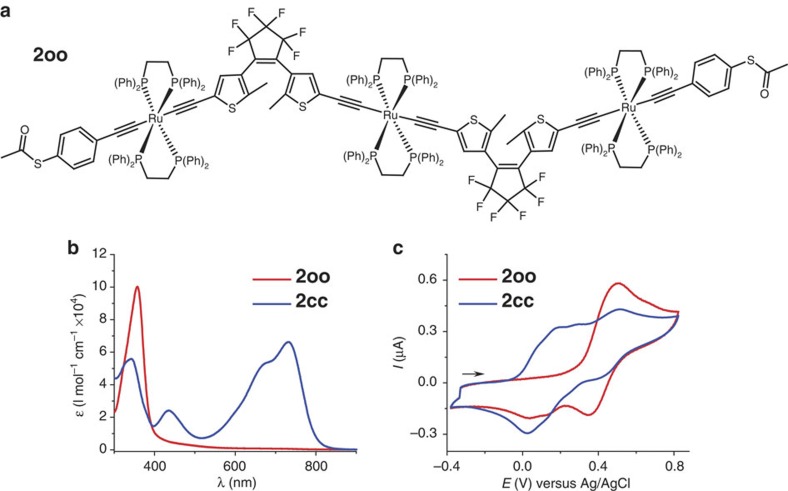
Orthogonally modulated isomerization of 2. (**a**) Chemical structure of 2oo. (**b**) Absorption spectra and spectral changes upon 350 nm irradiation of 2oo (50 μM in toluene). The process is fully reversible upon irradiation of 2cc at 750 nm. (**c**) CV of 2oo in CH_2_Cl_2_ (0.2 M Bu_4_NPF_6_) before (red line) and after irradiation at 350 nm leading to 2cc (blue line), scan rate 0.1 V s^−1^.

**Figure 5:**
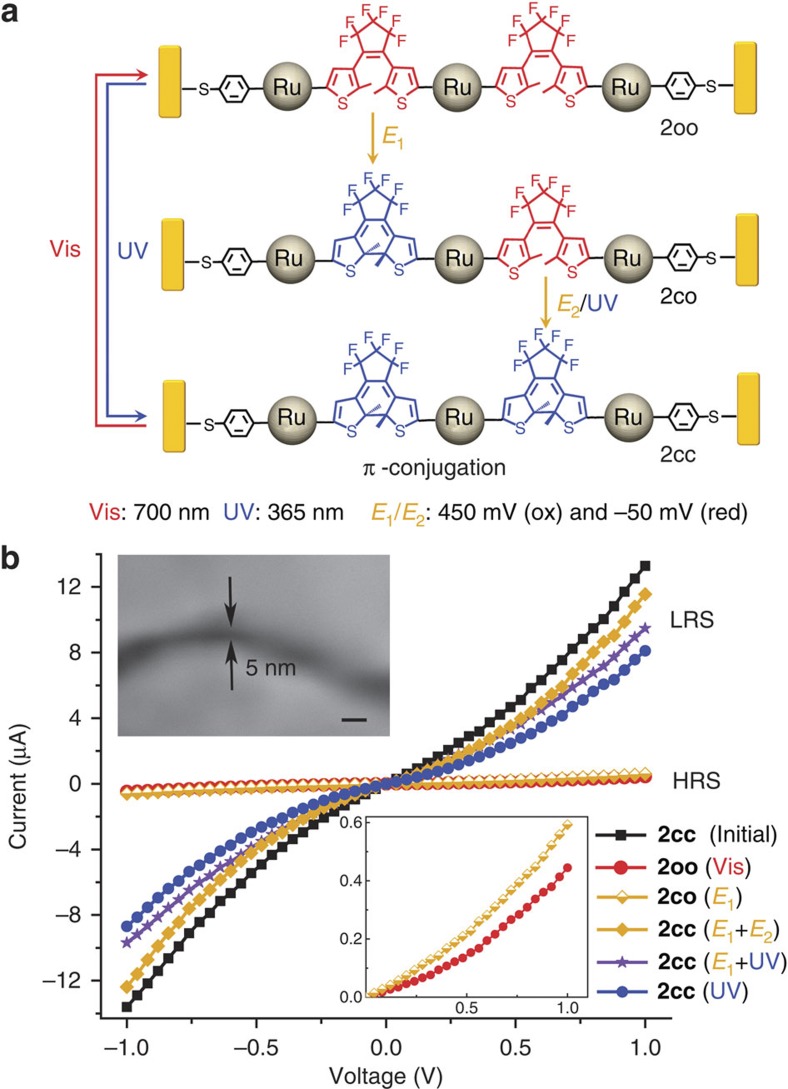
2cc-functionlized nanogap devices. (**a**) Scheme of molecular isomerization of **2** under external controls. E_1_ and E_2_, two cycles of electrolysis; UV, UV irradiation; Vis, visible light irradiation. (**b**) *I–V* characteristics of a ~5-nm gap device loaded with 2cc under vacuum. Black curve: initial 2cc device. Red curve: after 700 nm irradiation of 2cc for 2 h to 2oo device. Half yellow curve: the resulting **2co** device after one step of electrolysis of the 2oo device at 450 mV versus Ag/AgCl for 10 min followed by −50 mV for 10 min in CH_2_Cl_2_. Yellow curve: after two cycles of such electrolysis of 2oo to achieve the 2cc device. Purple curve: one electrolysis cycle of 2oo followed by 365 nm irradiation for 30 min to achieve the 2cc device. Blue curve: 365 nm irradiation of 2oo for 30 min to 2cc device. Inset: SEM image of ~5 nm gap (scale bar: 10 nm).

In order to explore more advanced functions toward multi-addressable light and electro-triggered multifunctional objects and to increase device complexity, the new trinuclear compound 2oo was designed, as is depicted in Fig. 4a. It was readily achieved using the similar synthetic pathway to that used for the previously reported analogue complex without the protected linking functions^41^. 2oo was fully characterized and shows characteristics similar to those of the latter. In particular, isomerization studies unambiguously show that the photochromic conversion of 2oo to the very stable 2cc complex upon irradiation is complete and fully reversible (Fig. 4b). More specifically, the colourless complex 2oo displays an absorption at *λ*_max_=357 nm, and upon irradiation with UV light (350 nm), this band vanishes while a broad absorption assigned to the deep green closed isomer 2cc appears at *λ*_max_=733 nm. This solution can be further bleached back to the colourless solution of 2oo under visible light (750 nm) via ring opening. During light irradiation, the intermediate **2co** is just transitorily formed and cannot be isolated, as already demonstrated with the related complex^41^. A typical CV of 2oo is shown in Fig. 4c that presents a similar wave to that of **1o** at *E*_pa_=500 mV versus Ag/AgCl composed of three slightly separated one-electron oxidation for the three electronically independent metal fragments leading to 2oo^3+^. During the reverse cathodic scan, two new waves appear at less positive potentials and are ascribed to the **2oc**^+^/**2oc** and **2oc**^2+^/**2oc**^+^ processes for the open–closed isomer electrochemically generated (Supplementary Fig. 3), since only one radical coupling could be obtained with oxidation of three Ru centres^41^. The CV of 2cc is obtained after photolysis (*λ*=350 nm) of 2oo, and presents four systems at *E°*_c1_=94 mV, *E°*_c2_=210 mV, *E°*_c3_=330 mV and *E°*_c4_=500 mV. Thus, as illustrated in Fig. 5a, 2oo displays the same unique properties as those of its parent compound without the linking functions, and is thereby a multi-addressable light and electro-triggered multifunctional object for integration in nanogaps. Of particular interest is the stepwise ring closure: after electrochemical oxidation followed by reduction to the neutral state, a single ring closure leads to **2oc**, and further full closure to 2cc can thus be accomplished electrochemically or with UV light^41^.

Complex 2cc was further assembled into OWL-generated ~5 nm gap devices (Fig. 5b, inset), under the same condition as those used for **1c** immobilization. The gap size here was tailored to be consistent with the length of 2cc that was estimated to ca. 49 Å´ using molecular model based on the bimetallic parent complex^40^,^44^. As shown in Fig. 5b, a characteristic *I–V* response has been observed with the symmetric black curve, which represented the successful formation of 2cc-based transport junctions. As observed with **1c**-based device, the conductivity of the 2cc-based nanodevices decreased after irradiating with 700 nm visible light (Fig. 5b, red curve), corresponding to the molecular isomerization from the fully π-conjugated 2cc (LRS) to the non-conjugated 2oo (HRS). Then, molecule **2** can also undergo the fully reverse photo-isomerization upon UV irradiation, and directly interchange from 2oo to 2cc through the transitory **2oc**. The device is then tuned back from HRS to LRS (Fig. 5b, blue curve).

More interestingly, further application of the electrochemical stimuli defined as *E*_1_ (sequentially applying 450 mV for oxidation of the organometallic ruthenium moieties, and −50 mV for reduction of the complex to the neutral state) to the 2oo-based nanodevice, to perform DTE ring closure as for **1o**-based devices, led to no significant change in the conductance of the device. Only a slight increase could be observed as shown by the half yellow curve in Fig. 5b. This expected fact is attributed to a single DTE electrochemical cyclization of 2oo to **2co**, since only one ring closure is obtained via one radical coupling in the triply oxidized molecule 2oo^3+^ to provide **2co**^3+^ before reduction, as stated above^41^. Thus, in this case, the non-conjugated open DTE unit dominates the resistance of the whole molecule and the device is maintained in the HRS state. Starting from this **2co** state, the conductivity jumps to the LRS when the device is further exposed to 365 nm UV irradiation (Fig. 5b, purple curve), as expected, leading to the 2cc-based nanodevice. Significantly, when the **2co**-device is further submitted to another electrochemical cycle (also sequentially applying 450 mV, and −50 mV), defined as *E*_2_, under the same conditions as the first (Fig. 5b, yellow curve), the LRS is also reached. These results indicated that two DTE units are fully closed and that the π-conjugated 2cc is also formed within the nanogap in both cases. Thus, in addition to the one-step UV irradiation, the full molecular isomerization of 2oo to 2cc in the nanogap can be also achieved by stepwise and orthogonal electrochemical and photo stimulations or via a two-step electrochemical process, most likely via two stepwise radical couplings of the two DTE units. It should be noted that owing to the unique gap structure of this nanodevice, the two opposite nanorods of the gap can both be employed as working electrodes for electrolysis, and therefore the later step can be achieved using the same or the opposite nanoelectrodes.

**Figure 6:**
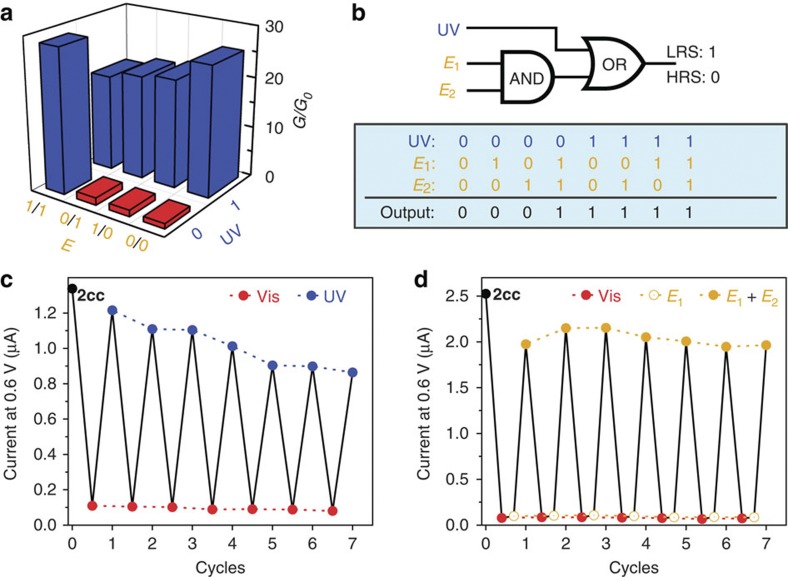
Resettable AND-OR logic gate based on 2cc-based MTJs. (**a**) Conductance (*G*) changes with different input combinations. (**b**) The electronic symbol and a truth table of the AND-OR logic gate. (**c**,**d**) Endurance performance: current response of a 2cc-based nanodevice under alternate modulation of visible light irradiation (700 nm for 2 h) and (**c**) UV irradiation (365 nm for 30 min) or (**d**) two successive electrochemical cycles (oxidation at 450 mV and reduction at −50 mV for 10 min). The current values were recorded at 0.6 V bias in vacuum.

As discussed with **1c**, 2cc-functionalized MTJs can also be designed for Boolean operation (Supplementary Fig. 4). Upon 700 nm light irradiation, the 2cc-functionalized device is set to the 2oo-based HRS. Electrochemical modulation and UV irradiation for ring closures can be employed as inputs. First, without introduction of 365 nm UV light irradiation (UV: 0), the presence and absence of each of the electrochemical input step (*E*_1_ and *E*_2_) were defined as ‘1’ and ‘0’. Only the presence of both inputs (1, 1) could cause the two stepwise ring closure to form π-conjugated pattern of 2cc, promoting the electron transport (output=1); while in absence of both (0, 0) or either inputs (1,0 or 0,1), the device conductivity remain as HRS (output=0) (Fig. 6a). These results suggest that an electronic AND gate has been achieved with only stepwise electrochemical modulations. It is worth noting that although the device remained in HRS with the inputs of (0, 0), (0, 1) or (1, 0), the molecular structure is not the same in all cases. By one-step electrochemical modulation, 2oo likely completed a single ring closure to form **2co**. As discussed above, in this case, only slight conductance enhancement was observed and the device remained in HRS. On the other hand, when the UV irradiation as the third terminal input (UV: 1) was used, 2oo could undergo the photo-isomerization to 2cc by closure of two DTE units simultaneously, and therefore the device were directly tuned from HRS to LRS (Fig. 6a), irrespective of any electrochemical stimuli being applied. Hence, a more complicated three-terminal logic gate has been developed combining the sequential Boolean computing AND and OR. (Fig. 6b)

Then, this functionalized three-terminal logic gate is also resettable with the help of 700-nm irradiation. Therefore, as shown in the Fig. 6c,d, both photo- and electro-triggered conductive switching could be repeatedly operated. Note that the progressively reduced reversibility in Fig. 6c also suggests that UV irradiation may cause decomposition of some functional molecules with time. In contrast, the stepwise electrochemical modulations clearly exhibit reversibility in Fig. 6d. This fact highlights the pertinence of our electrochemically driven ruthenium/DTE nanodevices. Note that owing to the sensitivity of UV irradiation, the stability of such logic devices is not yet competitive to conventional semiconductor devices. However this first proof-of-concept of MTJ-based logic devices will push forward the development of such multifunctional molecular devices.

## Discussion

It is worth noting that the yield of operative devices was ~21% (18/86 on a single chip of 1 cm × 1 cm, Supplementary Table 1) for **1c**-functionalized devices and 17% (13/77 on a single chip of 1 cm × 1 cm, Supplementary Table 2) for 2cc-functionalized devices. For different operative devices, a disparity in conductivity can be observed (current range: ~10^−5^ to ~10^−9^ A, at 1 V bias). This disparity should be mainly ascribed to the different number of functional molecules that actually span the nanogaps from one to the other^44^, which may be due to the ±0.2 nm gap size variation, and the uncontrollable roughness and morphology of the gold surface^51^,^52^,^53^. As estimated on the diameter of molecules^44^, up to ~3 × 10^4^ molecules can be ideally assembled in the junction (see experimental methods for detail), which is according to the four orders of magnitude range of the measured currents. As similar conductivity disparity can be found in the other reported molecular junctions based on the OWL-generated nanogap^51^,^52^,^53^, and considering the different size and resistance of single functional molecules, all these highly agreed with each other. In addition, the lowest observed current magnitude is at ~nA level that corresponds to the junction with only few molecules loaded, which is comparable to the results of single-molecule studies in previous reports on shorter DTE-containing molecules^13^,^34^. Besides the conductivity disparity, the current ratios between ‘1’ and ‘0’ states of different devices exhibit a slight fluctuation (Supplementary Tables 1 and 2). However, these ratios are all located in the range of 10–20 times, which are consistent with theoretical^44^ or experimental^13^,^34^ closed/open current ratios of single DTE derivatives, taking into account the high number of molecules loaded in the nanogaps. Such random fluctuation may be ascribed to slightly different orientations and conformations of functional molecules within each working device with variable roughness affecting the efficiency of the switching events. Note that the logic nanodevices presented here show a longer switching time compared with the traditional semiconductor systems, especially during photo-stimulation. This issue should be significantly improved through quantity control of assembled functional molecules within the junction since molecular number also determines the photostationary switching time upon light irradiation^64^, and amelioration of the device design for the irradiation procedure. Therefore, there is a lot of room for improvements in the optimization of device fabrication and functional molecule assembly to fit the potential application of complex logic computing in future studies.

In summary, we have demonstrated the achievement of multi-modulated MTJs based on the association of original ruthenium/DTE complexes and OWL-fabricated nanogaps with suitable gap sizes. The devices represent the very first molecular nanodevices that perform bi-state switchable conductivity by judicious control of the orthogonal light irradiation and electrochemical stimuli. The successive structural rearrangement of the incorporated molecules, that is, the photo-triggered reversible isomerization and especially the low-potential electro-triggered metal-promoted electrochemical cyclization, are all based on rational molecular design that allows stable Au–S bond connection between the functional molecules and two nanoelectrodes. Remarkably, the unique electronic structure of the longer molecule combining two DTE units offered the achievement of stepwise modulation. Therefore, depending on the properties of orthogonal modulation, these multi-addressable MTJ-based nanodevices can be designed to achieve Boolean computing, such as the presented OR operator and the three-input device AND-OR logic operation. Importantly, these organometallic molecular wire-based logic gates can be simply reset by introduction of visible light, ensuring the cycled set–reset operation. Although such logic computing cannot achieve logic flow through sequential gates so far, as the other chemical system-based approaches^57^, the proof-of-concept of logic gate demonstrated here still provides a solid platform for the development of multifunctional molecular devices after optimizing fabrication and operative conditions.

## Methods

### Synthesis and molecular studies

Reactions were all achieved by using the Schlenk techniques, under an inert atmosphere. Solvents were freshly distillated under argon using standard procedures. The synthesis of the binuclear compound, *trans*-[AcS–*p*–C_6_H_4_–C≡C–(dppe)_2_Ru–C≡C–(DTE)–C≡C–Ru(dppe)_2_–C≡C–*p*–C_6_H_4_–SAc] (**1o**), was previously reported^44^, and that of the new trinuclear compound, *trans*-[AcS–*p*–C_6_H_4_–C≡C–(dppe)_2_Ru–C≡C–(C_15_S_2_F_6_H_8_)–C≡C–Ru(dppe)_2_–C≡C–(C_15_S_2_F_6_H_8_)–C≡C–Ru(dppe)_2_–C≡C–*p*–C_6_H_4_–SAc] (2oo), was based on the precursors H–C≡C–(C_15_S_2_F_6_H_6_)–C≡C–Ru(dppe)_2_–C≡C–(C_15_S_2_F_6_H_6_)–C≡C–H (**3**)^41^ and [(dppe)_2_Ru=C=CHC_6_H_4_SAc](OTf) (**4**)^44^ (Supplementary Fig. 5). In one Schlenk tube, complex **3** (32 mg, 0.02 mmol) was dried under vacuum for 30 min before addition of dichloromethane (20 ml) and triethylamine (4.0 ml, 0.37 mmol). The solution was cannula-transferred into a second Schlenk tube containing the metal vinylidene **4** (54 mg, 0.04 mmol) and NaPF_6_ (12 mg, 0.07 mmol), also previously dried under vacuum for 30 minutes. The reaction mixture was stirred at room temperature for 5 days. Then, the reaction mixture was washed with water (3 × 15 ml). The remaining solvent was removed under reduced pressure. The residue was taken up in dichloromethane (10 ml), and pentane (30 ml) was slowly added leading to the formation of a light brown precipitate that was washed with further pentane (2 × 15 ml) to obtain 2oo (60 mg, 81%). Data for 2oo: ^1^H NMR (500 MHz, C_6_D_6_, 297 K) *δ*: 7.73–6.92 (*m*, 128 H, C_6_H_5_+C_6_H_4_-p-SCOCH_3_), 6.83 (*s*, 2H, H _DTE_), 6.74 (*s*, 2H, H _DTE_), 2.47 (*m*, 24H, PCH_2_CH_2_P), 2.00 (*s*, 12H, CH_3 DTE_) 1.92 (*s*, 6H, COCH_3_). ^31^P NMR (121 MHz, C_6_D_6_, 297 K) *δ*: 53.5 (*s*, dppe_remote_), 53.4 (*s*, dppe_central_). IR (KBr): ν=1,702 cm^−1^ (C=O), 2052, cm^−1^ (C≡C). HR-MS FAB^+^ 3872.5555 (*m/z*): ([M]^+.^, calcd 3,872.5623). Analysis for C_214_H_174_F_12_O_2_P_12_Ru_3_S_6_,: C 66.69, H 4.43 (cacld: C 66.37, H 4.53). (Supplementary Fig. 6) 2cc was then obtained after excitation at *λ*=350 nm in the NMR tube. Data for 2cc: ^1^H NMR (500 MHz, CD_3_Cl_3_, 297 K) *δ*: 7.36–6.91 (*m*, 124H C_6_H_5_+o-C_6_H_4_-p-SCOCH_3_), 6.81 (*d*, ^3^J_HH_=6.0 Hz, 4H, m-C_6_H_4_-p-SCOCH_3_), 6.34 (*s*, 2H, H_DTE_), 6.24 (*s*, 2H, H_DTE_), 2.59 (*m*, 24H, PCH_2_CH_2_P), 2.42 (*s*, 6H, COCH_3_), 1.82 (*s*, 6H, CH_3 DTE_), 1.81 (*s*, 6H, CH_3 DTE_). ^31^P NMR (121 MHz, CDCl_3_, 297 K) *δ*: 53.0 (*s*, dppe_remote_), 52.4 (*s*, dppe_central_). (Supplementary Fig. 7)

High-resolution mass spectra (HR-MS) were recorded on a ZabSpecTOF (LSIMS at 4 kV) spectrometer. UV–vis irradiations were performed in toluene with a LS series Light Source of ABET Technologies, Inc (150 W xenon lamp), equipped with single wavelength light filters ‘350FS 10–25’ and ‘750FS 40–25’. UV–vis-NIR spectra were recorded with a Cary 5000 apparatus. Electrochemical studies were carried out under argon using an Autolab PGSTAT 30 potentiostat (CH_2_Cl_2_, 0.2 M Bu_4_NPF_6_). The working electrode was a Pt disk and ferrocene the internal reference. It should be noted that all the reactions and handling of the compounds were carried out in the dark.

### Device fabrication

Gold nanowires (~5 μm) with specific nanogaps (~3 nm, and ~5 nm corresponding to the actual length of **1** and **2**) were prepared following the standard procedures of OWL^45^,^46^,^47^. Au-Ni-Au multi-segment nanowires were firstly obtained by electrochemical deposition of commercial electroplating solutions (Ag: 1025 Silver, Ni: Nickel Sulphamate RTU and Au: Orotemp 24RTU, Technic Inc.) within an anodic aluminium oxide (AAO) porous membrane (Whatman, Anodisc 0.02 μm pores, 47 mm outer diameter) that was previously coated with 200 nm Ag layer on the reverse, as both the template and the working electrode. Ag was deposited as an initial electrical contact layer under DC current at −800 mV (versus Ag/AgCl), while nickel was plated at −850 mV and gold was plated at −950 mV to form nanowires. The thickness of the sacrificed layer (Ni) was charge-controlled by 10 mC for 1 nm. After Ag contact layer and AAO template were sequentially dissolved, plasma-enhanced chemical vapour deposition was used to deposit ~50 nm silica on the wire surface. Finally, the sacrificial layer of Ni was etched by 1 M HCl for 3 h, and then a ~3 nm or a ~5 nm gap was generated in the 5-μm nanorod for the assembly of functional molecules.

The outer gold microeletrodes were patterned via standard processes of photolithography on a Si/SiO_2_ chip. Then, after being deposited onto a chip with patterned gold microeletrodes, the two ends of the fabricated nanowire were connected to microelectrodes using e-beam lithography and sequential metal deposition of 5 nm Cr and 400 nm Au. (See Supplementary Methods for details). SEM (JEOL JSM-7600F) was employed to observe the morphology of the nanodevices and to measure the size of the nanogap.

### Functional molecule assembly

The fabricated nanogap devices were cleaned using oxygen plasma for 5 min. The wafer was then immersed in the 5 ml degassed THF solution containing 1 mg **1c** or 2cc in the dark for 24 h, and under N_2_ atmosphere. This THF solution of **1c** or 2cc was obtained by the following method: (1) The THF solution of precursor open state molecules, **1o** or 2oo, was irradiated with 365 nm UV light for 30 min to ensure that all the open molecules switched to closed state; (2) the degassed solution of **1c** or 2cc NH_4_OH (28% of NH_3_, 5 μl) was added dropwise for deprotection of the thiol. The wafer with nanogap devices was further rinsed with THF and ethanol, and then dried with N_2_. It should be noted that the more rigid closed state molecules were preferred to the unfavourable molecular conformation of the open state molecules (less linear and rigid) to form active MTJs (Supplementary Fig. 8)^44^. As the gold nanorod is of 300 nm in diameter, the accessible surface is thus roughly of 7 × 10^4^ nm^2^ if the surface is considered as planar (minimum surface). The molecules have a diameter of 1.5 nm (ref. 44), if grafted perpendicularly to the electrode surface, thus roughly of 2.25 nm^2^ in surface. Therefore, a maximum number of ~3 × 10^4^ molecules could be ideally assembled in the junction.

### External stimulations

UV–vis irradiation was performed with a 150 W xenon lamp with single wavelength light filters at 365 nm (30 min) and 700 nm (2 h). All electrochemical stimuli were performed using an electrochemical analyser, model 832C (CH Instruments). Electrolysis for DTE cyclization were carried at 0.45 V (10 min) for oxidation and −0.05 V (10 min) for reduction in CH_2_Cl_2_ with a three-electrode system consisting of the gold nanoelectrode as working electrode, a platinum wire counter electrode and a Ag/AgCl reference electrode.

### Electrical measurements

The current–voltage characteristics of the multi-modulated nanodevices were obtained in solid state using a semiconductor parameter analyser (Keithley 4200-SCS) for the application of a potential and measurement of currents, combined with a cryogen-free micromanipulated probe station (Janis CCR-12) for connecting the microelectrodes. Unless otherwise stated, the measurements were conducted under vacuum (1 × 10^−4^ torr).

## Author contributions

X.C. and S.R. proposed the concept. F.M., S.R. and X.C. designed the experiment. Y.-M.H., L.N. and S.R. synthesized the molecules. F.M., Q.S. and B.H. fabricated the nanodevices. F.M., S.R., Y.-M.H., L.N. and X.C. discussed the results, and co-wrote the manuscript.

## Additional information

**How to cite this article:** Meng, F. *et al*. Orthogonally modulated molecular transport junctions for resettable electronic logic gates. *Nat. Commun.* 5:3023 doi: 10.1038/ncomms4023 (2014).

## Supplementary Material

Supplementary Information Supplementary Figures 1-8, Supplementary Tables 1-2 and Supplementary Methods
