# Supplementary material for: Orthogonally modulated molecular transport junctions for resettable electronic logic gates
Source: Nat Commun. 2014 Jan 7;5:3023. doi: 10.1038/ncomms4023 (PMC3896775; doi:10.1038/ncomms4023)
Supplement: Supplementary Information — Supplementary Figures 1-8, Supplementary Tables 1-2 and Supplementary Methods [file ncomms4023-s1.pdf]

Supplementary Information

Orthogonally Modulated Molecular Transport Junctions for Resettable Electronic Logic Gates

Fanben Meng,[1] Yves-Marie Hervault,[2] Qi Shao,[1] Benhui Hu,[1] Lucie Norel,[2] Stéphane Rigaut,[2] and Xiaodong Chen[1]

[1]School of Materials Science and Engineering, Nanyang Technological University, 50 Nanyang Avenue, Singapore 639798, Singapore.
[2]Institut des Sciences Chimique de Rennes, UMR 6226 CNRS – Université de Rennes 1, 263 Av. du Général Leclerc, F-35042 Rennes cedex, France
Correspondence and requests for materials should be addressed to X.C. (email: chenxd@ntu.edu.sg) or to S.R. (email: stephane.rigaut@univ-rennes1.fr).

Supplementary Figures:

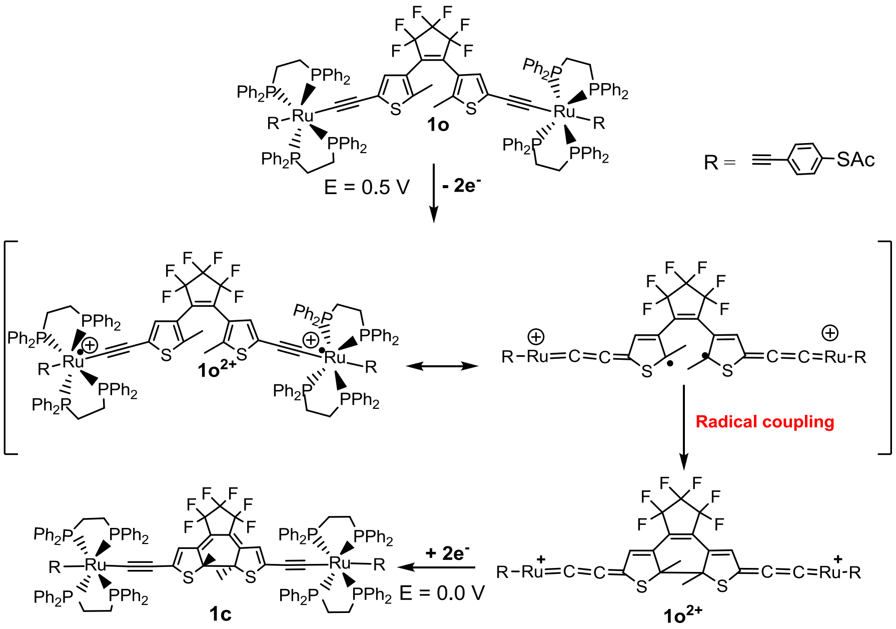

Supplementary Figure 1 | ** Schematic electro-cyclisation of **1o.

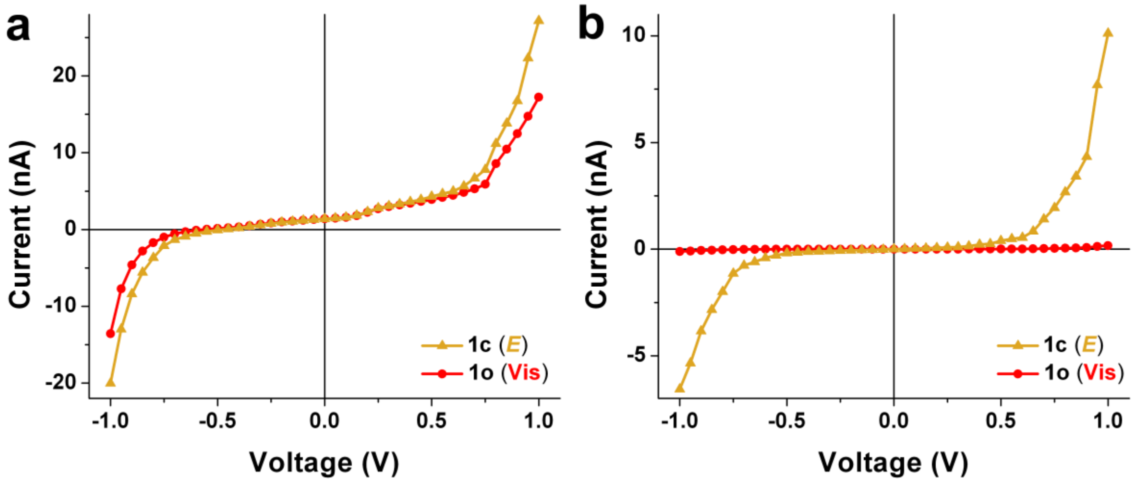

Supplementary Figure 2 | Representative *I-V* characteristics of **1c**-functionalized ~3 nm OWL-generated gap devices (a) in CH_2Cl_2 solution or (b) in Vacuum.

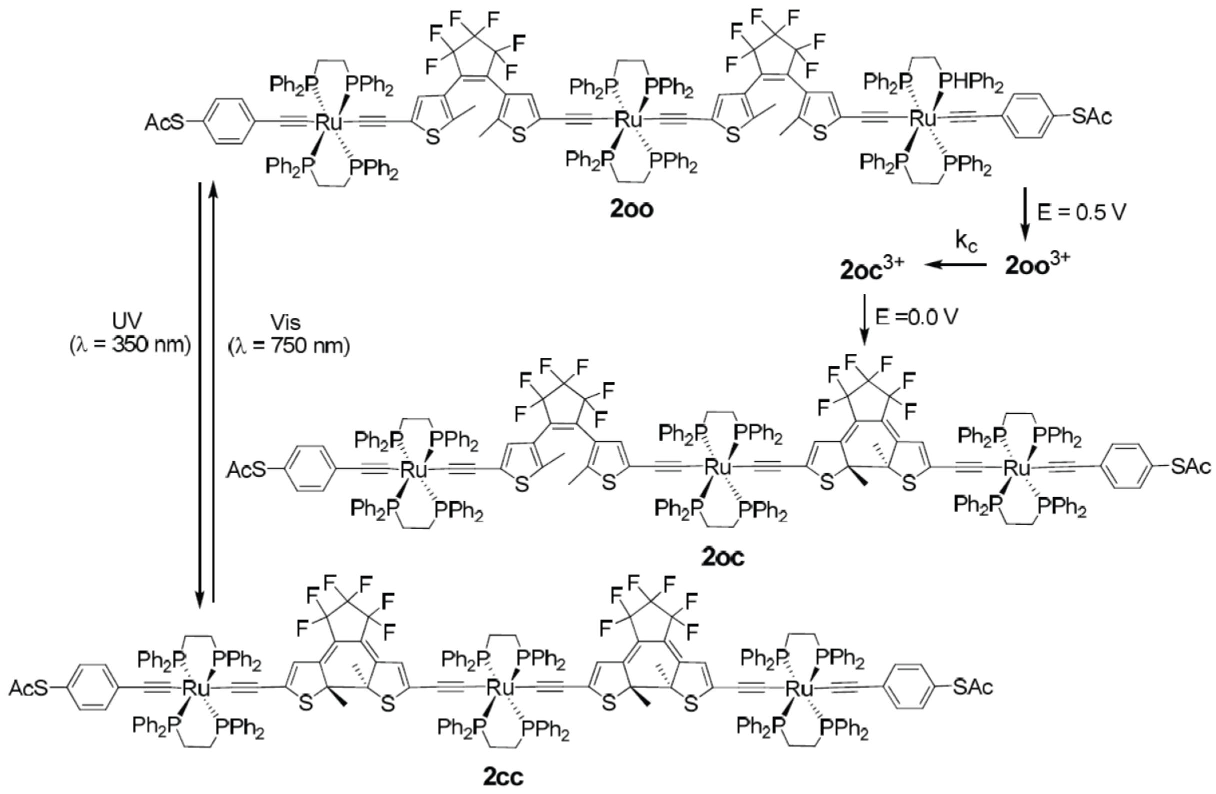

Supplementary Figure 3 │ Schematic isomerization of **2**.

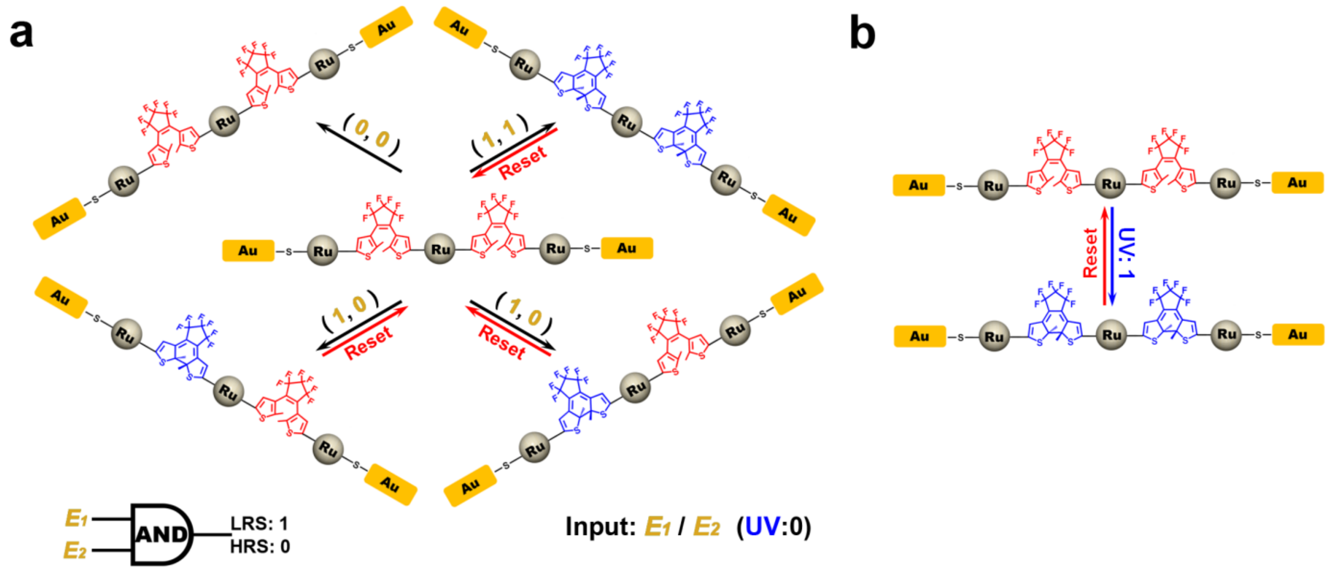

Supplementary Figure 4 | Scheme of **2**-based logic gate (a) without or (b) with UV irradiation. (Reset: visible light irradiation)

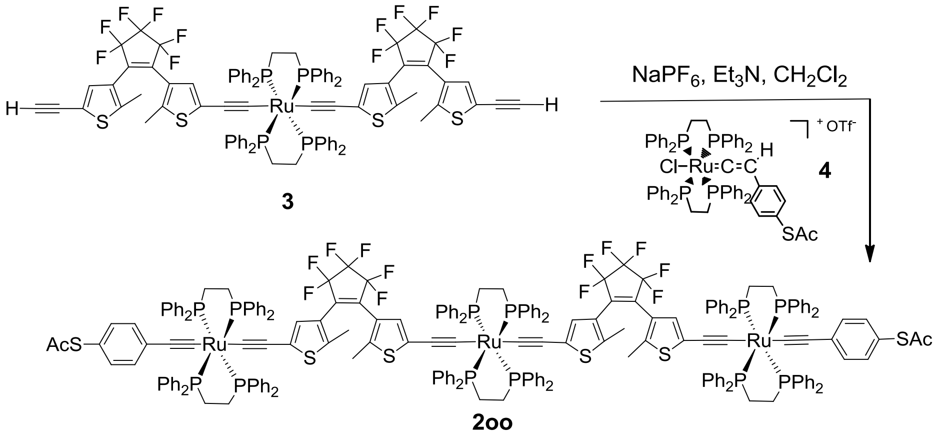

Supplementary Figure 5 │ Scheme of synthesis process of **2oo**.

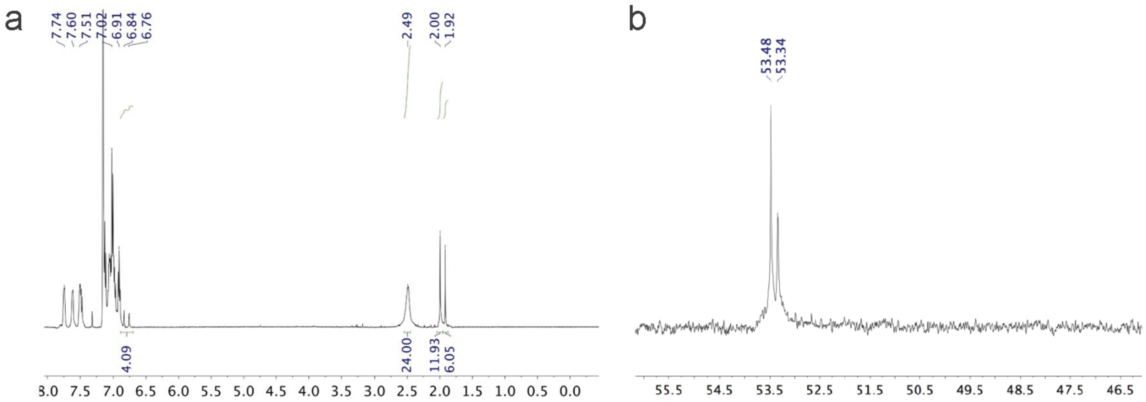

Supplementary Figure 6 │ (a) ^{1}H and (b) ^{31}P NMR spectrum of **2oo** in C_6D_6.

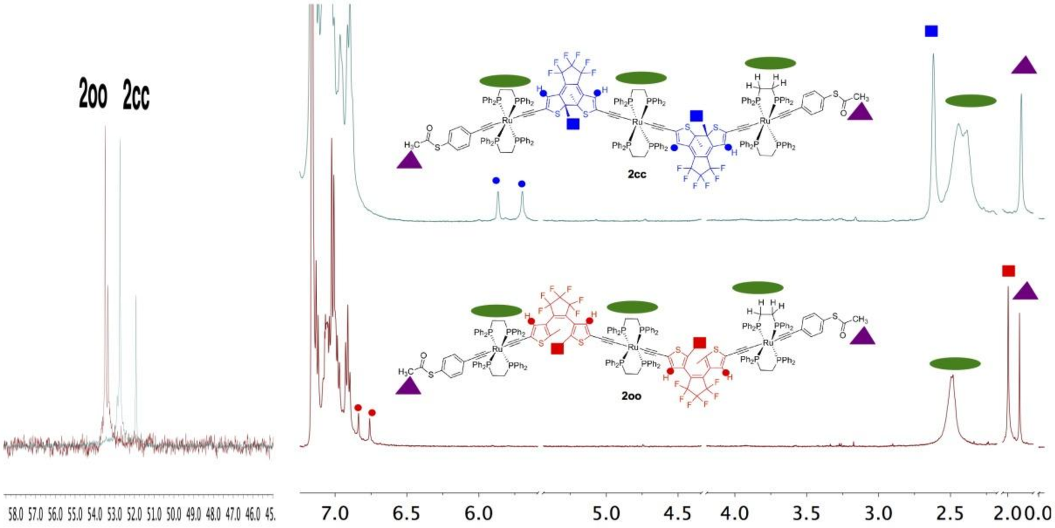

Supplementary Figure 7 │ Selected ^{31}P NMR (left) and ^{1}H NMR (right) signals in C_6D_6 of **2oo** and of **2cc** after excitation at λ = 350 nm in the NMR tube. Initial spectra were recovered after bleaching at λ = 750 nm.

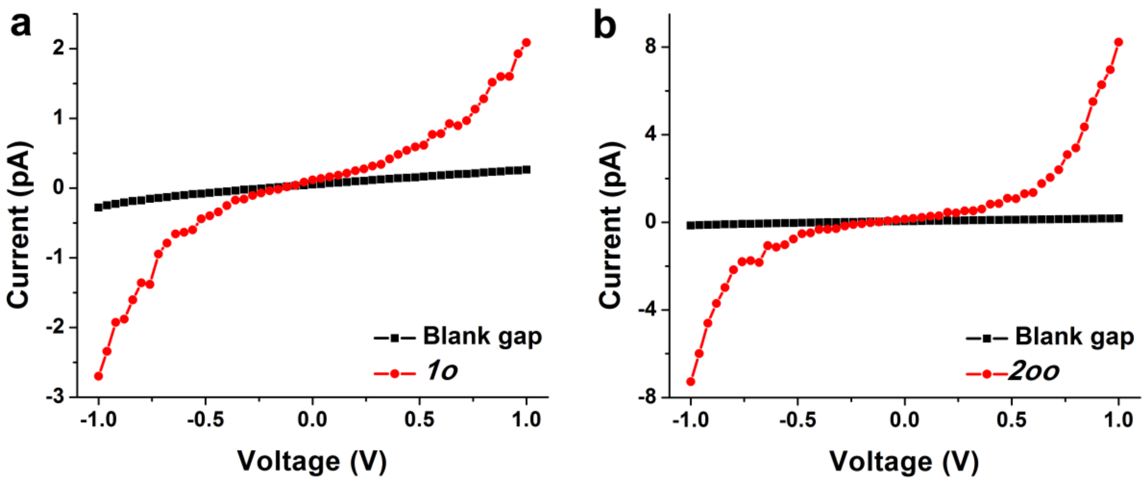

Supplementary Figure 8 | Representative *I*-*V* characteristics of (a) blank (black curve) and **1o**-fabricated (red curve) ~3 nm OWL-generated gap devices and (b) blank (black curve) and **2oo**-fabricated (red curve) ~5 nm OWL-generated gap devices.

Supplementary Tables:

Supplementary Table 1. 1c-based nanodevices.

Devices	1	2	3	4	5	6	7	8	9	10	11	12	13	14	15	16	17	18
**1c** (initial)	57.9 (μA)	22.1 (μA)	16.7 (μA)	11.6 (μA)	8.93 (μA)	5.78 (μA)	5.12 (μA)	2.63 (μA)	2.01 (μA)	1.76 (μA)	0.83 (μA)	0.39 (μA)	79.2 (nA)	28.4 (nA)	10.2 (nA)	7.71 (nA)	1.85 (nA)	0.82 (nA)
**1o** (Vis)	2.17 (μA)	1.79 (μA)	0.58 (μA)	0.49 (μA)	0.60 (μA)	0.37 (μA)	0.67 (μA)	0.14 (μA)	0.32 (μA)	93.6 (nA)	30.1 (nA)	24.7 (nA)	4.77 (nA)	3.48 (nA)	1.92 (nA)	0.52 (nA)	176 (pA)	90.0 (pA)
**1c** (*E*)	44.4 (μA)	20.5 (μA)	14.0 (μA)	10.8 (μA)	6.68 (μA)	5.03 (μA)	4.55 (μA)	2.02 (μA)	1.72 (μA)	1.47 (μA)	0.69 (μA)	0.29 (μA)	71.3 (nA)	22.6 (nA)	8.70 (nA)	7.44 (nA)	1.50 (nA)	0.54 (nA)
**1c** (UV)	35.9 (μA)	18.7 (μA)	12.3 (μA)	9.12 (μA)	6.11 (μA)	3.78 (μA)	3.61 (μA)	1.57 (μA)	1.59 (μA)	1.29 (μA)	0.54 (μA)	0.26 (μA)	60.5 (nA)	18.4 (nA)	5.69 (nA)	5.95 (nA)	1.28 (nA)	0.49 (nA)

[a] Current values were recorded at 0.6 V.

Supplementary Table 2. 2cc-based nanodevices.

Devices	1	2	3	4	5	6	7	8	9	10	11	12	13
**2cc** (initial)	20.8 (μA)	12.2 (μA)	9.07 (μA)	6.31 (μA)	4.39 (μA)	2.52 (μA)	1.34 (μA)	0.51 (μA)	0.37 (μA)	117.6 (nA)	30.2 (nA)	8.48 (nA)	1.13 (nA)
**2oo** (Vis)	0.93 (μA)	0.54 (μA)	0.51 (μA)	0.19 (μA)	0.47 (μA)	84.8 (nA)	90.4 (nA)	27.2 (nA)	15.6 (nA)	9.88 (nA)	4.97 (nA)	1.93 (nA)	0.21 (nA)
**2co** (E_1)	1.12 (μA)	0.72 (μA)	0.58 (μA)	0.28 (μA)	0.49 (μA)	107.8 (nA)	110.1 (nA)	30.9 (nA)	16.6 (nA)	11.3 (nA)	5.04 (nA)	1.90 (nA)	0.22 (nA)
**2cc** ($E_1 + E_2$)	18.5 (μA)	10.6 (μA)	8.92 (μA)	4.94 (μA)	3.61 (μA)	2.15 (μA)	1.22 (μA)	0.39 (nA)	0.32 (nA)	110.4 (nA)	22.1 (nA)	6.29 (nA)	0.85 (nA)
**2cc** (E_1 + UV)	16.1 (μA)	9.33 (μA)	7.02 (μA)	4.50 (μA)	3.07 (μA)	1.89 (μA)	1.07 (μA)	0.32 (nA)	0.26 (nA)	100.9 (nA)	16.9 (nA)	5.98 (nA)	0.69 (nA)
**2cc** (UV)	13.8 (μA)	7.14 (μA)	5.99 (μA)	3.47 (μA)	2.54 (μA)	1.66 (μA)	0.90 (μA)	0.27 (nA)	0.21 (nA)	89.7 (nA)	13.2 (nA)	5.13 (nA)	0.44 (nA)

[a] Current values were recorded at 0.6 V.

Supplementary Methods:

Device fabrication. First, microelectrodes were formed via photolithography in three steps. 1) Si wafers with 600 nm SiO_2 layer were cleaned via sonication in acetone and ethanol for 30 min, rinsed with ethanol and dried with N_2, and then placed in an oven (95 ℃) for 5 min after spin coating with a photoresist (AZ 1518 Photoresist, Shipley, USA) at 500 rpm for 5s and 4500 rpm for 45 s. 2) The resist was subsequently patterned using a mask aligner (SUSS MJB4 Mask Aligner, Garching, Germany) and developed with AZ 300 MIF. 3) Cr (4 nm) and Au (50 nm) were thermally evaporated onto the patterned wafer and the wafer was immersed in acetone for liftoff. Subsequently, a suspension of gold nanorods with OWL-fabricated nanogaps was deposited on a chip containing prefabricated Au microelectrodes. Electron beam lithography (EBL) was employed to define an inner electrode pattern that connects the nanorods with the microelectrodes. A resist layer of PMMA was prepared by the following procedure: 950 PMMA C7 was spin-coated at 500 rpm (for 10 s) and 3000 rpm (for 45 s) followed by baking at 180 ℃ for 4 min. EBL was carried out using a SEM (JEOL JSM-6360 SEM, Tokyo, Japan), equipped with the Raith SEM lithography kits (Raith ELPHY Quantum, Dortmund, Germany) at 30 kV acceleration voltage and 40 pA beam current. Cr (5 nm) and Au (400 nm) were then thermally evaporated onto the e-beam resist-coated substrate after it had been developed with 1:3 (v/v) MIBK/IPA solution for 30 s, and then rinsed with IPA. Finally, the two ends of the nanorod were connected with the microelectrodes. These nanogap devices were ready for assembling functional molecules, and for electrical measurement.
